# Supplementary material for: Absence of Heme Oxygenase-1 Affects Trophoblastic Spheroid Implantation and Provokes Dysregulation of Stress and Angiogenesis Gene Expression in the Uterus
Source: Cells. 2024 Feb 22;13(5):376. doi: 10.3390/cells13050376 (PMC10930528; doi:10.3390/cells13050376)
Supplement: Supplementary file 1 [file cells-13-00376-s001.zip › cells-2844005-supplementary.pdf]

SUPPLEMENTARY INFORMATION

Absence of heme oxygenase-1 affects blastocyst implantation and provokes a dysregulation of stress and angiogenesis gene expression in the uterus

Authors: Maria Laura Zenclussen [1,*], Sina Ulrich [2], Mario Bauer [3], Beate Fink [3], Ana Claudia Zenclussen [2,3,4], Anne Schumacher [2,3,4,*,†], Nicole Meyer [2,3,4,*,†]

[1] Instituto de Salud y Ambiente del Litoral (ISAL, UNL-CONICET), Facultad de Bioquímica y Ciencias. Biológicas, Universidad Nacional del Litoral (UNL), Santa Fe 3000, Argentina

[2] Experimental Obstretrics and Gynecology, Medical Faculty, Otto-von-Guericke University Magdeburg, 39108 Magdeburg, Germany

[3] Department of Environmental Immunology, Helmholtz Centre for Environmental Research-UFZ, 04318 Leipzig, Germany

[4] Perinatal Immunology, Saxonian Incubator for Clinical Translation (SIKT), Medical Faculty, Leipzig University, 04103 Leipzig, Germany

***** Correspondence: mzenclussen@gmail.com (M.L.Z.); nicole.meyer@ufz.de (N.M.); Tel.: +49-341-6025-1542 (N.M.)

[†] These authors contributed equally to this work.

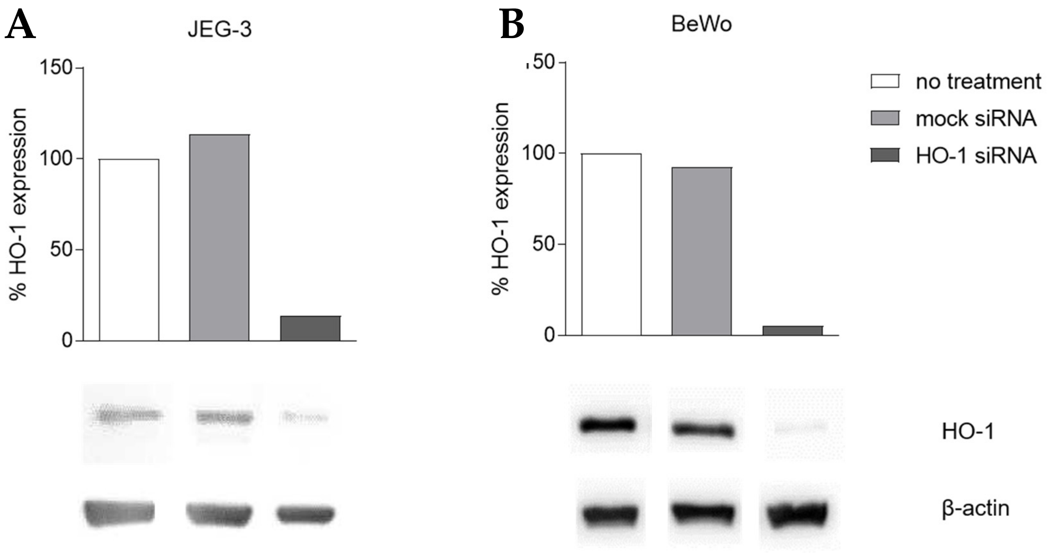

Figure S1: Western Blot analysis of the knock-down of HO-1 expression by siRNA. Western Blot analysis of the knock-down of HO-1 expression by siRNA in JEG-3 (A) and in BeWo (B) blastocysts. Mock siRNA: cells transfected with 0.2 nM Silencer® Select Negative Control siRNA; HO-1 siRNA: cells transfected with 0.2 nM Silencer® Select HO-1 siRNA.

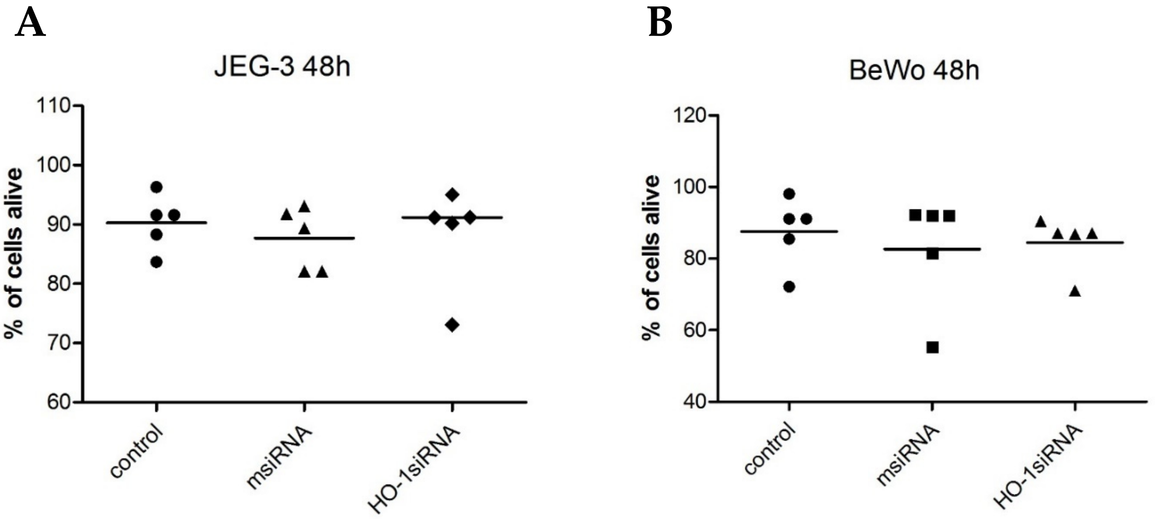

Figure S2: Viability of spheroids after 48h of culture. Viability of JEG-3 (A) or BeWo (B) spheroids after 48 h of culture. Control: cells without treatment. msiRNA: cells transfected with 0.2 nM Silencer® Select Negative Control siRNA; HO-1 siRNA: cells transfected with 0.2 nM Silencer® Select HO-1 siRNA.

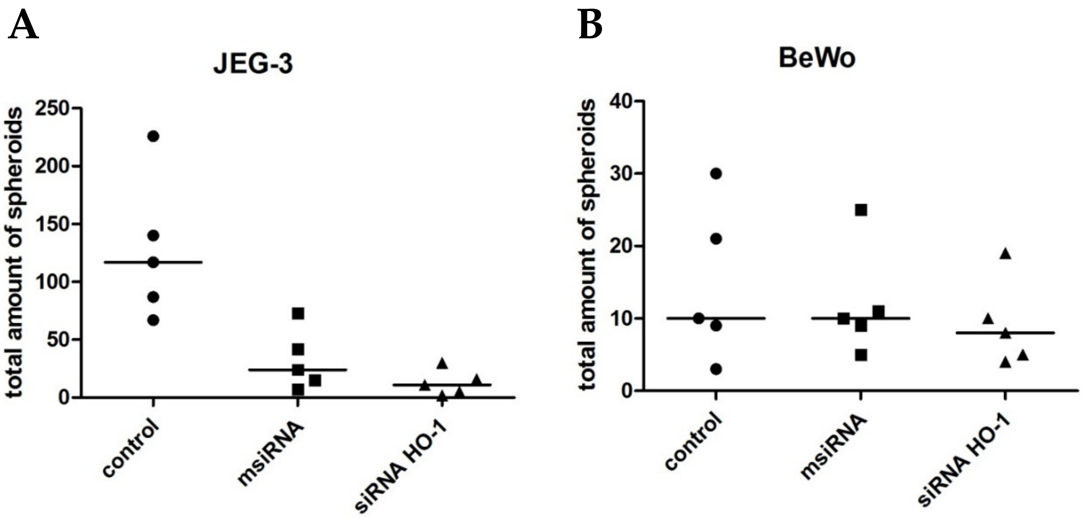

Figure S3: Total number of spheroids. Total number of spheroids of JEG-3 trophoblasts (A) or BeWo trophoblasts (B). Control: cells without treatment. msiRNA: cells transfected with 0.2 nM Silencer® Select Negative Control siRNA; HO-1 siRNA: cells transfected with 0.2 nM Silencer® Select HO-1 siRNA.

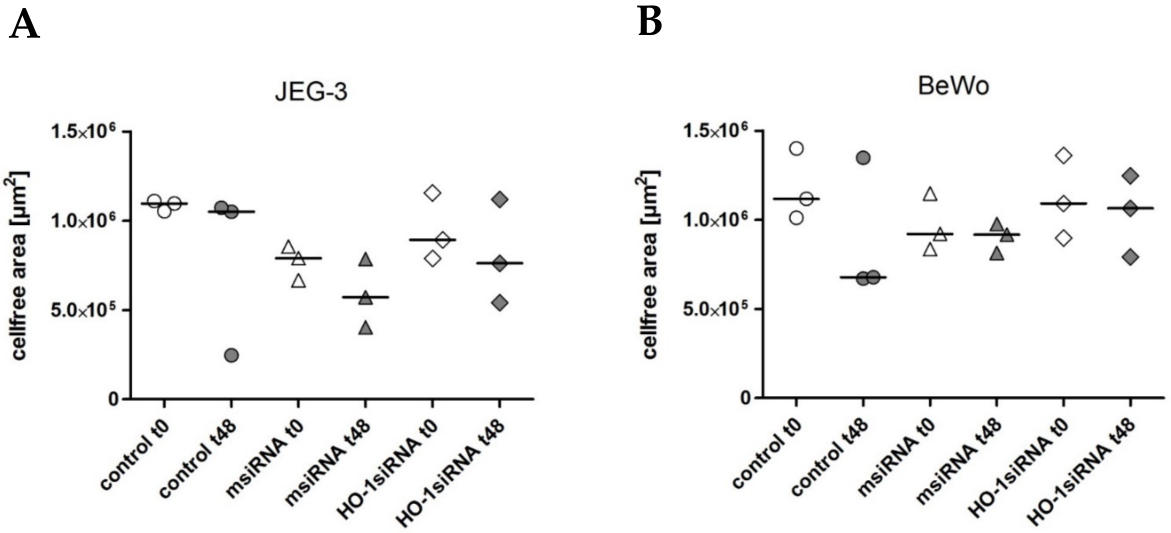

Figure S4: Wound healing assay to assess trophoblast migration. Cell free area in a wound healing assay to assess trophoblast migration using JEG-3 trophoblasts (A) or BeWo trophoblasts (B). Control: cells without treatment. msiRNA: cells transfected with 0.2 nM Silencer® Select Negative Control siRNA; HO-1 siRNA: cells transfected with 0.2 nM Silencer® Select HO-1 siRNA. t0= initial time (creation of the wound); t48: 48 hours after wound healing.

Table S1: Mouse Angiogenesis PCR Array RT2-Profiler™ PCR Array. Symbol and name of the genes involved in angiogenesis as well as five reference genes (actin, beta, *Actb*; glyceraldehyde-3-phosphate dehydrogenase, *Gapdh*; heat shock protein 90kDa alpha (cytosolic), class B member 1, *Hsp90ab1*; hypoxanthine guanine phosphoribosyl transferase 1, *Hprt1*; and glucuronidase, beta, *Gusb*), as analyzed by the Mouse Angiogenesis RT2-Profiler™ PCR Array.

Symbol	Description	Symbol	Description
Angpt1	Angiopoietin 1	*Lep*	Leptin
Angpt2	Angiopoietin 2	*Mapk14*	Mitogen activated protein kinase 14
Anpep	Alanyl (membrane) aminopeptidase	*Mdk*	Midkine
Bai1	Brain-specific angiogenesis inhibitor 1	*Mmp19*	Matrix metallopeptidase 19
Ccl11	Small chemokine (C-C motif) ligand 11	*Mmp2*	Matrix metallopeptidase 2
Ccl2	Chemokine (C-C motif) ligand 2	*Mmp9*	Matrix metallopeptidase 9
Cdh5	Cadherin 5	*Npr1*	Natriuretic peptide receptor 1
Col18a1	Procollagen, type XVIII, alpha 1	*Nrp1*	Neuropilin 1
Col4a3	Procollagen, type IV, alpha 3	*Nrp2*	Neuropilin 2
Csf3	Colony stimulating factor 3 (granulocyte)	*Pdgfa*	Platelet derived growth factor, alpha
Ctgf	Connective tissue growth factor	*Pecam1*	Platelet/endothelial cell adhesion molecule 1
Cxcl1	Chemokine (C-X-C motif) ligand 1	*Pgf*	Placental growth factor
Cxcl2	Chemokine (C-X-C motif) ligand 2	*Plau*	Plasminogen activator, urokinase
Cxcl5	Chemokine (C-X-C motif) ligand 5	*Plg*	Plasminogen
Ecgf1	Endothelial cell growth factor 1 (platelet-derived)	*Plxdc1*	Plexin domain containing 1
Edg1	Endothelial differentiation sphingolipid G-protein-coupled receptor 1	*Ptgs1*	Prostaglandin-endoperoxide synthase 1
Efna1	Ephrin A1	*Serpinf1*	Serine (or cysteine) peptidase inhibitor, clade F, member 1
Efnb2	Ephrin B2	*Smad5*	MAD homolog 5 (Drosophila)
Egf	Epidermal growth factor	*Sphk1*	Sphingosine kinase 1
Eng	Endoglin	*Stab1*	Stabilin 1
Epas1	Endothelial PAS domain protein 1	*Tbx1*	T-box 1
Ephb4	Eph receptor B4	*Tbx4*	T-box 4
Ereg	Epiregulin	*Tek*	Endothelial-specific receptor tyrosine kinase
F2	Coagulation factor II	*Tgfa*	Transforming growth factor alpha
Fgf1	Fibroblast growth factor 1	*Tgfb1*	Transforming growth factor, beta 1
Fgf2	Fibroblast growth factor 2	*Tgfb2*	Transforming growth factor, beta 2
Fgf6	Fibroblast growth factor 6	*Tgfb3*	Transforming growth factor, beta 3
Fgfr3	Fibroblast growth factor receptor 3	*Tgfbr1*	Transforming growth factor, beta receptor I
Figf	C-fos induced growth factor	*Thbs1*	Thrombospondin 1
Flt1	FMS-like tyrosine kinase 1	*Thbs2*	Thrombospondin 2
Fzd5	Frizzled homolog 5 (Drosophila)	*Timp1*	Tissue inhibitor of metalloproteinase 1
Gna13	Guanine nucleotide binding protein, alpha 13	*Timp2*	Tissue inhibitor of metalloproteinase 2
Hand2	Heart and neural crest derivatives expressed transcript 2	*Tmprss6*	Transmembrane serine protease 6
Hgf	Hepatocyte growth factor	*Tnf*	Tumor necrosis factor
Hif1a	Hypoxia inducible factor 1, alpha subunit	*Tnfaip2*	Tumor necrosis factor, alpha-induced protein 2
Ifng	Interferon gamma	*Tnfsf12*	Tumor necrosis factor (ligand) superfamily, member 12
Igf1	Insulin-like growth factor 1	*Vegfa*	Vascular endothelial growth factor A
Il1b	Interleukin 1 beta	*Vegfb*	Vascular endothelial growth factor B
Il6	Interleukin 6	*Vegfc*	Vascular endothelial growth factor C
Itgav	Integrin alpha V	*Gusb*	Glucuronidase, beta
Itgb3	Integrin beta 3	*Hprt1*	Hypoxanthine guanine phosphoribosyl transferase 1
Jag1	Jagged 1	*Hsp90ab1*	Heat shock protein 90kDa alpha (cytosolic), class B member 1
Kdr	Kinase insert domain protein receptor	*Gapdh*	Glyceraldehyde-3-phosphate dehydrogenase
Lama5	Laminin, alpha 5	*Actb*	Actin, beta, cytoplasmic
Lect1	Leukocyte cell derived chemotaxin 1		

Table S2: Mouse Stress and Toxicity PathwayFinder™ RT2 Profiler™ PCR Array. Symbol and name of the genes whose expression level is indicative of stress and toxicity as well as five reference genes (actin, beta, *Actb;* glyceraldehyde-3-phosphate dehydrogenase, *Gapdh;* heat shock protein 90kDa alpha (cytosolic), class B member 1, *Hsp90ab1;* hypoxanthine guanine phosphoribosyl transferase 1, *Hprt1;* and glucuronidase, beta, *Gusb*), as analyzed by the Mouse Stress and Toxicity PathwayFinder™ RT2 Profiler™ PCR Array.

Symbol	Description	Symbol	Description
Anxa5	Annexin A5	Hmox2	Heme oxygenase (decycling) 2
Atm	Ataxia telangiectasia mutated homolog (human)	Hsf1	Heat shock factor 1
Bax	Bcl2-associated X protein	Hspa1b	Heat shock protein 1B
Bcl2l1	Bcl2-like 1	Hspa1l	Heat shock protein 1-like
Casp1	Caspase 1	Hspa4	Heat shock protein 4
Casp8	Caspase 8	Hspa5	Heat shock 70kD protein 5 (glucose-regulated protein)
Ccl21b	Chemokine (C-C motif) ligand 21b	Hspa8	Heat shock protein 8
Ccl3	Chemokine (C-C motif) ligand 3	Hspb1	Heat shock protein 1
Ccl4	Chemokine (C-C motif) ligand 4	Hspd1	Heat shock protein 1 (chaperonin)
Ccnc	Cyclin C	Hspe1	Heat shock protein 1 (chaperonin 10)
Ccnd1	Cyclin D1	Igfbp6	Insulin-like growth factor binding protein 6
Ccng1	Cyclin G1	Il18	Interleukin 18
Cdkn1a	Cyclin-dependent kinase inhibitor 1A (P21)	Il1a	Interleukin 1 alpha
Chek2	CHK2 checkpoint homolog (S. pombe)	Il1b	Interleukin 1 beta
Cryab	Crystallin, alpha B	Il6	Interleukin 6
Csf2	Colony stimulating factor 2 (granulocyte-macrophage)	Lta	Lymphotoxin A
Cxcl10	Chemokine (C-X-C motif) ligand 10	Mdm2	Transformed mouse 3T3 cell double minute 2
Cyp1a1	Cytochrome P450, family 1, subfamily a, polypeptide 1	Mif	Macrophage migration inhibitory factor
Cyp1b1	Cytochrome P450, family 1, subfamily b, polypeptide 1	Mt2	Metallothionein 2
Cyp2a5	Cytochrome P450, family 2, subfamily a, polypeptide 5	Nfkb1	Nuclear factor of kappa light chain gene enhancer in B-cells 1, p105
Cyp2b10	Cytochrome P450, family 2, subfamily b, polypeptide 10	Nfkbia	Nuclear factor of kappa light chain gene enhancer in B-cells inhibitor, alpha
Cyp2b9	Cytochrome P450, family 2, subfamily b, polypeptide 9	Nos2	Nitric oxide synthase 2, inducible, macrophage
Cyp2c29	Cytochrome P450, family 2, subfamily c, polypeptide 29	Pcna	Proliferating cell nuclear antigen
Cyp3a11	Cytochrome P450, family 3, subfamily a, polypeptide 11	Polr2k	Polymerase (RNA) II (DNA directed) polypeptide K
Cyp4a10	Cytochrome P450, family 4, subfamily a, polypeptide 10	Por	P450 (cytochrome) oxidoreductase
Cyp4a14	Cytochrome P450, family 4, subfamily a, polypeptide 14	Rad23a	RAD23a homolog (S. cerevisiae)
Cyp7a1	Cytochrome P450, family 7, subfamily a, polypeptide 1	Rad50	RAD50 homolog (S. cerevisiae)
Ddit3	DNA-damage inducible transcript 3	*Serpine1*	Serine (or cysteine) peptidase inhibitor, clade E, member 1
Dnaja1	DnaJ (Hsp40) homolog, subfamily A, member 1	*Sod1*	Superoxide dismutase 1, soluble
E2f1	E2F transcription factor 1	*Sod2*	Superoxide dismutase 2, mitocondrial
Egr1	Early growth response 1	*Tnfrsf1a*	Tumor necrosis factor receptor superfamily, member 1a
Ephx2	Epoxide hydrolase 2, cytoplasmic	*Tnfsf10*	Tumor necrosis factor (ligand) superfamily, member 10

Ercc1	Excision repair cross-complementing rodent repair deficiency, complementation group 1	*Tradd*	TNFRSF1A-associated via death domain
Ercc4	Excision repair cross-complementing rodent repair deficiency, complementation group 4	*Trp53*	Transformation related protein 53
Fasl	Fas ligand (TNF superfamily, member 6)	*Ugt1a2*	UDP glucuronosyltransferase 1 family, polypeptide A2
Fmo1	Flavin containing monooxygenase 1	*Ung*	Uracil DNA glycosylase
Fmo4	Flavin containing monooxygenase 4	*Xrcc1*	X-ray repair complementing defective repair in Chinese hamster cells 1
Fmo5	Flavin containing monooxygenase 5	*Xrcc2*	X-ray repair complementing defective repair in Chinese hamster cells 2
Gadd45a	Growth arrest and DNA-damage-inducible 45 alpha	*Xrcc4*	X-ray repair complementing defective repair in Chinese hamster cells 4
Gpx1	Glutathione peroxidase 1	*Gusb*	Glucuronidase, beta
Gpx2	Glutathione peroxidase 2	*Hprt1*	Hypoxanthine guanine phosphoribosyl transferase 1
Gsr	Glutathione reductase 1	*Hsp90ab1*	Heat shock protein 90kDa alpha (cytosolic), class B member 1
Gstm1	Glutathione S-transferase, mu 1	*Gapdh*	Glyceraldehyde-3-phosphate dehydrogenase
Gstm3	Glutathione S-transferase, mu 3	*Actb*	Actin, beta, cytoplasmic
Hmox1	Heme oxygenase (decycling) 1		

Table S3: RT-qPCR Primers. UPL, Universal Probe Library probe

Gene	Forward Primer	Reverse Primer	UPL Number
Actb	aaggccaaccgtgaaaagat	gtggtacgaccagaggcatac	56
Ccl1	gcttcccctgaagtttatcca	aggcgcagctttctctacct	63
Ccl11	agagctccacagcgcttct	gcaggaagttgggatgga	18
Ccl17	tgcttctggggacttttctg	gaatggccccttgaagtaa	27
Ccl2	gtccctgtcatgcttctgg	cgttaactgcatctggctga	19
Ccl20	aactgggtgaaaagggctgt	gtccaattccatcccaaaaa	73
Ccl3	ttgaaaccagcagcctttg	gccggtttctcttagtcagga	20
Ccl4	cctctctctcctcttgctcgt	ggagggtcagagcccatt	1
Ccl5	tgcagaggactctgagacagc	gagtggtgtccgagccata	110
Csf2	gcatgtagaggccatcaaaga	cgggtctgcacacatgtta	79
Cxcl1	gactccagccacactccaac	tgacagcgcagctcattg	83
Cxcl5	ttcttgggtgtgttaagagtgttc	tctgcatgacacagcagctt	26
Cxcl10	gctgccgtcattttctgc	tctcactggcccgtcatc	18
Cxcl12	ccaaactgtgcccttcagat	atttcgggtcaatgcacact	41
Cxcl13	cagaatgaggctcagcacag	atgggcttccagaataccg	63
Fasl	accggtggtatttttcatgg	tttaaggctttggttggtgaa	21
Gapdh	gggttcctataaatacggactgc	ccattttgtctacgggacga	52
Hmox	gacacctgaggtcaagcaca	tcctctgtcagcatcacctg	15
Ifnb1	actgcctttgccatccaa	cccagtgctggagaaattgt	50
Ifng	cattcagagctgcagtgacc	ctgtctggcctgctgttaaa	52
Il11	tactccgccgtttacagctc	cagggggatcacaggttg	27
Il12a	ccaggtgtcttagccagtcc	gcagtgcaggaataatgtttca	62
Il13	acccagaggatattgcatgg	tgggctacttcgattttggt	19
Il1a	ttggttaaatgacctgcaaca	gagcgctcacgaacagttg	52
IL1b	agttgacggaccccaaaag	tttgaagctggatgctctcat	26
IL27	catggcatcacctctctgac	aagggccgaagtgtggta	38
Il2rb	tcgacacaactccatgttgc	cagggaagagctatggtagcc	17
Il3	cgtctctcctaaccgtggaa	gccatgaggaacattcagact	13
Il4	ggtctcaacccccagctagt	tggatatggctcctggtacat	15
Il5	acattgaccgccaaaaagag	atccaggaactgcctcgtc	91
Il6	tgatggatgctaccaaactgg	ttcatgtactccaggtagctatgg	6
Mmp2	aactttgagaaggatggcaagt	tgccacccatggtaaacaa	29

Mmp9	acgacatagacggcatcca	gctgtggttcagttgtggtg	19
Ntrk2	attctgcctgctggtgatgt	ctccagaatacctcttttcctctct	15
Rplp0	ctgctgaacatgctgaacatc	tgtcgagcacttcagggtta	62
Runx1	ctccgtgctacccactcact	atgacggtgaccagagtgc	77
Serpine1	aggatcgaggtaaacgagagc	gcgggctgagatgacaaa	69
Sod1	ccatcagtatggggacaataca	ggtctccaacatgcctctct	49
Sod2	tgctctaatcaggacccattg	gtagtaagcgtgctcccacac	3
Tgfb1	gcaacatgtggaactctaccag	cagccactcaggcgtatca	66
Timp1	gcaaagagctttctcaaagacc	agggatagataaacagggaaacact	76
Timp2	ttttgcaatgcagacgtagtg	ggaatccacctccttctcg	21
Tnfsf10	tgagaacctttcaggacacca	gagctgccactttctgaggt	52
Trp53	acgcttctccgaagactgg	agggagctcgaggctgata	25
Ubc	gtctgctgtgtgaggactgc	cctccagggtgatggtctta	77
Vegfa	actggaccctggctttactg	tctgctctccttctgtcgtg	22
